# Identification of *ECI2* as Potential Prognostic Biomarkers Based on a Fatty Acid Metabolism-Related Gene Model in Clear Cell Renal Cell Carcinoma

**DOI:** 10.1155/genr/2237539

**Published:** 2025-05-19

**Authors:** Di Cui, Wenye Yang, Bo Guan, Wenxu Wu, Wenjiang Yu

**Affiliations:** ^1^Fuyang Medical College, Fuyang Normal University, Fuyang 236037, Anhui, China; ^2^Clinical Laboratory, Fuyang Minsheng Hospital, Fuyang 236072, Anhui, China; ^3^Urology Surgery, Fuyang People's Hospital, Fuyang 236000, Anhui, China; ^4^Department of Health Examination Center, First People's Hospital of Nanning, Nanning 530022, Guangxi, China; ^5^Thoracic and Cardiovascular Surgery, Fuyang Fifth People's Hospital, Fuyang 236037, Anhui, China

**Keywords:** clear cell renal cell carcinoma, *ECI2*, fatty acid metabolism

## Abstract

**Background:** Clear cell renal cell carcinoma (ccRCC) is the most common and highly malignant subtype of renal cancer, characterized by significant lipid deposition. Research has indicated that its growth and metastasis are closely associated with fatty acid metabolism.

**Methods:** In this study, we integrated TCGA transcriptome data, CPTAC proteomics data, and the single-cell dataset GSE152938 to identify differentially expressed genes related to fatty acid metabolism in ccRCC. Using the LASSO algorithm, we constructed a prognostic model based on these genes. Western blot and PCR analyses confirmed the expression levels of the *ECI2* in ccRCC, while lentiviral transduction was used to investigate the effects of *ECI2* expression on tumor biological behaviors.

**Results:** Our findings demonstrated that *ECI2* expression is downregulated in ccRCC, and lower *ECI2* levels correlate with better patient prognosis. Functional assays showed that overexpression of *ECI2* significantly inhibited the proliferation and migration of ccRCC cells and increased their sensitivity to the chemotherapeutic drug oxaliplatin.

**Conclusion:** This study highlights the potential tumor-suppressive role of *ECI2* in ccRCC and suggests its viability as a diagnostic and therapeutic target.

## 1. Introduction

Renal cell carcinoma (RCC) is a malignant tumor that arises from renal tubular epithelial cells [1]. Clear cell RCC (ccRCC) accounts for 80% to 90% of RCC and is the most common and aggressive type with the poorest prognosis [2, 3]. The incidence rate in males is 1.5 to 2.0 times that in females, with a peak age of incidence between 60 and 70 years. Statistics indicate that worldwide, there are over 330,000 new cases of RCC annually, leading to more than 140,000 deaths each year, with its incidence rate having consistently increased over recent decades [4]. The prognosis for advanced ccRCC is poor, with a 5-year survival rate of only 11.7% [5]. Because ccRCC is insensitive to both radiotherapy and chemotherapy, surgical removal remains the primary treatment for ccRCC [6].

Dysregulation of lipid metabolism is a widespread characteristic phenotype of cancer, with different tumor types displaying unique metabolic adaptations that facilitate the reprogramming of lipid metabolism within tumor cells [7, 8]. Research has shown that ccRCC demonstrates a significant inclination towards lipid deposition over lipid breakdown, with its proliferation and metastasis closely linked to fatty acid synthesis, cholesterol uptake, and transport [9]. The extensive lipid storage in ccRCC not only influences the tumor's energy homeostasis but also releases lipid substances during cell proliferation for the synthesis of cellular biomembranes [10]. Additionally, lipid accumulation plays a pivotal role in signal transduction and activity, proinflammatory responses, phenotype switching, and therapeutic resistance within ccRCC tumor cells [11]. *ECI2* is a gene related to lipid metabolism that encodes peroxisome D3, D2-enynyl CoA isomerase. It is commonly associated with endothelial cells and is involved in lipid metabolism–related signaling pathways [12]. The ECI2 protein, located on peroxisomes and mitochondria, plays a crucial role in lipid processing and metabolism [13]. Our study developed a new prognostic model related to lipid metabolism and investigated the functional impact of ECI2 on ccRCC cells, offering new targets and a theoretical foundation for the diagnosis and treatment of ccRCC.

## 2. Materials and Methods

### 2.1. Data Acquisition

The single-cell data is sourced from GSE152938, which includes four tumor samples and one normal kidney sample [14]. The regular transcriptome data is sourced from TCGA (https://portal.gdc.com). Protein data is sourced from renal clear cell carcinoma in CPTAC (https://proteomics.cancer.gov/programs/cptac). Fatty acid metabolism–related genes are downloaded from MSigDB database (https://www.gsea-msigdb.org/gsea/index.jsp), which contains a total of 158 genes.

### 2.2. Single-Cell Data Processing

We performed a comprehensive analysis using the “Seurat” and “SingleR” packages [15, 16]. To ensure data quality, we implemented three strict filtering criteria: retaining only genes expressed in at least five cells, removing cells with less than 100 expressed genes, and removing cells with mitochondrial gene expression exceeding 5%. Subsequently, using the “NormalizeData” function in the “Seurat” package, the data was standardized using the “LogNormalize” method. Perform cell clustering using the “FindClusters” function, employ the “FindAllMarkers” function, and combine Wilcoxon–Mann–Whitney test to identify differentially expressed genes in each cluster. This process sets strict filtering criteria, including adjusted *p* values less than 0.01 and |log_2_ (fold change)| greater than 1, to ensure the reliability of the results.

### 2.3. Construction of Prognostic Model

Clinical data from TCGA were used to perform Kaplan–Meier (KM) survival analysis, with the log-rank test applied to assess survival differences among groups. To evaluate the predictive accuracy of the model, the “timeROC” package was employed. Feature selection was conducted using the Least Absolute Shrinkage and Selection Operator (LASSO) regression algorithm implemented in the “glmnet” package, enabling the identification of key predictive variables [17]. The “survival” package was applied for multivariate Cox regression analysis to construct a prognostic model [18]. An optimal model was selected by combining multivariate Cox regression with iterative analysis via the step function, ensuring the most predictive and parsimonious model as the final choice.

### 2.4. Gene Function Enrichment Analysis

To further validate the potential role of the target gene, TCGA patients were stratified into high- and low-expression groups based on target gene expression levels. Differentially expressed genes were identified using the “Limma” package and subsequently analyzed through functional enrichment. Gene ontology (GO) analysis was employed to annotate gene functions, providing insights into biological processes, cellular components, and molecular functions associated with the target gene [19]. KEGG enrichment analysis serves as a useful tool for exploring gene functions and associated high-level genomic information. The “ClusterProfiler” package facilitated the analysis of potential mRNA GO functions and KEGG pathway enrichment [20].

### 2.5. Immune Infiltration Analysis

The relative abundance of immune cell infiltration in each sample is indicated by enrichment scores calculated through ssGSEA analysis, which labels each type of infiltrating immune cell. A heatmap was generated using the “pheatmap” package to visualize the immune cell infiltration distribution across samples. The TIDE approach assesses tumor immune evasion by analyzing gene expression markers, focusing on cytotoxic T lymphocytes (CTLs) dysfunction and their exclusion by immunosuppressive factors [21]. A high TIDE score is linked to unfavorable outcomes from immune checkpoint blockade (ICB) therapy and reduced survival post-treatment. We employed the TIDE algorithm to forecast *ECI2*'s response to immunotherapy.

### 2.6. Receiver Operating Characteristic (ROC) Curve and KM Survival Curve

After obtaining ccRCC expression data from TCGA, ROC analysis was performed on the data using “pROC” package to detect the specificity of *ECI2*. The results were visualized using “ggplot2” package. For the KM survival curve, the “survival” package was used for proportional hazards hypothesis testing and survival regression fitting, and the results were visualized using the “survminer” package and “ggplot2” package.

### 2.7. Sample Acquisition

The experimental subjects of this study were ccRCC patients of Fuyang People's Hospital. During the study, 48 pairs of tumor tissue samples and adjacent tissue samples (48 cancer, 48 adjacent cancer) were collected from patients undergoing ccRCC radical surgery. Among them, 40 pairs were used for immunohistochemical staining, and 8 pairs were used for real-time quantitative polymerase chain reaction (RT-PCR) and Western blot experiments. After obtaining the tissue sample through surgery, rinse it immediately with physiological saline and store it in a liquid nitrogen tank filled with liquid nitrogen. All samples were diagnosed with ccRCC through clinical and pathological diagnosis, and the research protocol meets the requirements of the Ethics Committee of Fuyang People's Hospital.

### 2.8. Cell Culture

Human-derived ccRCC cell lines (OS-RC-2, 769P, 786-O) and normal renal tubular epithelial cell line (HK-2) were used, all of which were purchased from ServiceBio. Inoculate the cells into 1640 medium containing 10% fetal bovine serum and culture them in a cell culture incubator at 37°C and 5% CO_2_. Change the fresh culture medium every 2–3 days and perform subculture.

### 2.9. Virus Transfection

Cells are seeded in a 24-well plate, and transfection reagents are added. According to the MOI and virus titer of the cells, the corresponding virus amount is added, and the cells are cultured at 37°C for 12–16 h. After replacing the complete culture medium and continuing to culture for about 72 h after infection, the cells are further cultured in a culture medium containing an appropriate concentration of puromycin for stable strain screening.

### 2.10. Immunohistochemical Staining

Mark the tissue edges with an immunohistochemical pen, add 3% hydrogen peroxide to block endogenous enzymes, and wash with PBS. Subsequently, add primary antibody dropwise onto the slices and incubate overnight at 4°C, then rinse with PBS. Add polymer enhancer and incubate at room temperature, wash with PBS, drip enzyme labeled polymer and incubate again, use fresh DAB for color development, and control the time under a microscope to avoid background coloring. Finally, hematoxylin counterstaining, hydrochloric acid alcohol differentiation, and tap water anti-blue were performed to complete the sealing process.

### 2.11. Immunofluorescence Staining

Take logarithmic growth phase cells and fix them with 4% paraformaldehyde for 10 min, then treat them with 5% Triton for 5 min. Then, mitochondrial morphology was labeled with TOM20 and incubated overnight at 4°C with 1 μg/mL primary antibody (1:200). The cells were incubated with fluorescent secondary antibody (1:1000) for 1 h and counterstained with DAPI.

### 2.12. Protein Extraction and Western Blot

Collect cells in logarithmic growth phase, extract proteins using RIPA lysis buffer containing 1% PMSF, and then detect protein concentration using an enhanced BCA assay kit. The obtained protein samples were electrophoretized on 10% SDS polyacrylamide gel and then transferred to PVDF membrane by electroblotting. Incubate primary and secondary antibodies sequentially to detect differences in the expression levels of relevant proteins.

### 2.13. Quantitative Real - time Reverse Transcription - Polymerase Chain Reaction (qRT-PCR)

The RNA extraction method and PCR quantitative analysis method are the same as our previous research [22]. The primers used for amplifying *ECI2* were: 5′-CCAGGCTCCACCTGA CTAGA-3′ and 5′-GGCCACTGAAGGACCTTGTA-3′. For *ACTB* amplification, the primers were 5′-TGGGACGACATGGAGAAAAT-3′ and 5′-AGAGGCGTACAGGGATAGCA-3′ (Tongyong Biotech, China).

### 2.14. Clone Formation

Seed cells in the logarithmic growth phase at roughly 1000 cells per well into a 6-well plate and incubate for 7–10 days until colonies become visible. After discarding the culture medium, wash the cells with PBS and fix them with methanol for 30 min. Stain each well with 1 mL of crystal violet for 15 min, then observe and count the colonies under a microscope.

### 2.15. Scratch Experiment

Cells were plated into each well of a 6-well plate, and a scratch was created using a 1 mL pipette tip. Afterward, the cells were rinsed three times with PBS and incubated in serum-free medium. Samples were collected at 0 and 24 h, and cell migration was observed and imaged at specified areas using an inverted microscope.

### 2.16. Transwell Experiment

Starve the cells in serum-free culture medium for 24 h before inoculation, then digest and collect the cells and count them. Add the cell suspension to the upper chamber of the Transwell chamber, add serum containing culture medium to the lower chamber, let it stand, and incubate for 48 h. Remove the small chamber, clean and fix it, stain it, remove the nonmigrating cells in the upper chamber, and finally observe and record the cell migration under a microscope.

### 2.17. Statistical Methods

R 4.2.1 software was used for analysis, and “ggplot2” package was used for visualization. Group *t* test or Mann–Whitney *U* test was used to compare the expression difference between ccRCC cancer tissues and adjacent tissues. *p* < 0.05 means the difference is statistically significant.

## 3. Results

### 3.1. Screening of Differentially Expressed Genes Related to Fatty Acid Metabolism (FA-DEGs)

**Figure 1:**
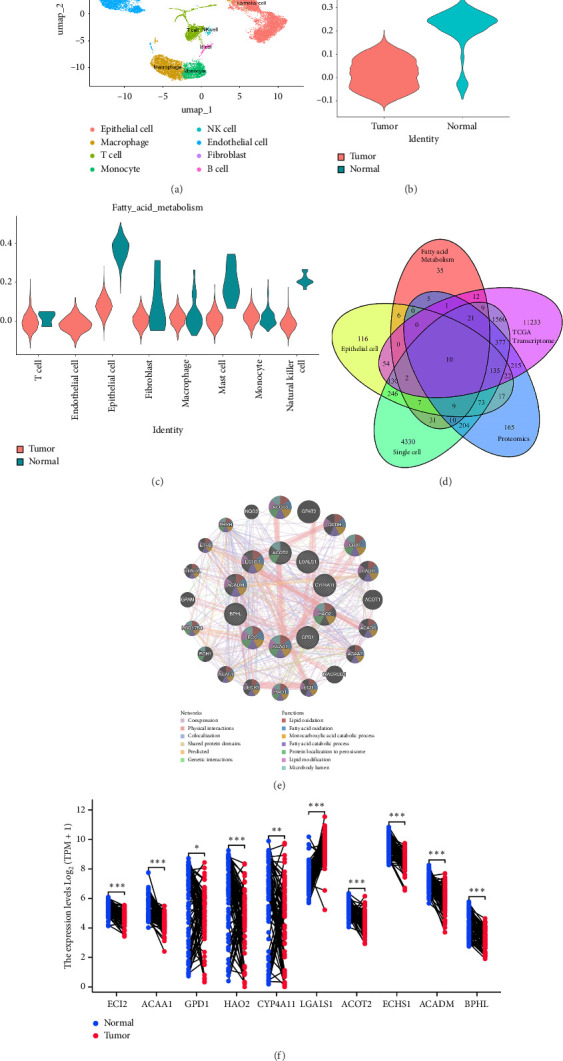
Screening of differentially expressed genes related to fatty acid metabolism. (a) Single-cell data clustering ump plot of GSE152938. (b) Differential expression of fatty acid metabolism gene set between tumor and adjacent tissues of ccRCC. (c) Differential expression of fatty acid metabolism gene set among different cell types. (d) Combining transcriptomics, proteomics, and single-cell genomics to screen FA-DEGs. (e) Protein interaction networks of 10 FA-DEGs using GENEMIA database. (f) Differential expression of 10 FA-DEGs in TCGA transcriptome. Data were shown as mean ± SD. ^∗^*p* < 0.05, ^∗∗^*p* < 0.01, ^∗∗∗^*p* < 0.001, ^∗∗∗∗^*p* < 0.0001.

We first performed cluster analysis on single-cell data and obtained seven cell populations including epithelial cell, macrophage, T cell, monocyte, NK cell, endothelial cell, and fibroblast and B cell (Figure 1(a)). Using the fatty acid metabolism gene set to score the expression of all cell types, it was found that epithelial cells exhibited the greatest differences (Figure 1(b)). Consistent with the overall score, the expression score of the fatty acid metabolism gene set is higher in normal tissues (Figure 1(c)). Next, we will take the intersection of the fatty acid gene set, TCGA transcriptome differentially expressed genes, single-cell population differentially expressed genes, epithelial cell differentially expressed genes, and proteomic differentially expressed genes (Figure 1(d)). Ten genes, including *ECI2, ACAA1, GPD1, HAO2, CYP4A11, LGALS1, ACOT2, ECHS1, ACADM*, and *BPHL*, have been named as FA-DEGs. A molecular interaction network was constructed for FA-DEGs using GENEMIA database, with molecular functions focused on lipid oxidation, fatty acid oxidation (FAO), and monocarboxylic acid catabolic process (Figure 1(e)). Differential analysis showed that nine genes were upregulated and one gene was downregulated in tumor tissues (Figure 1(f)).

### 3.2. Prognostic Model Related to Fatty Acid Metabolism

**Figure 2:**
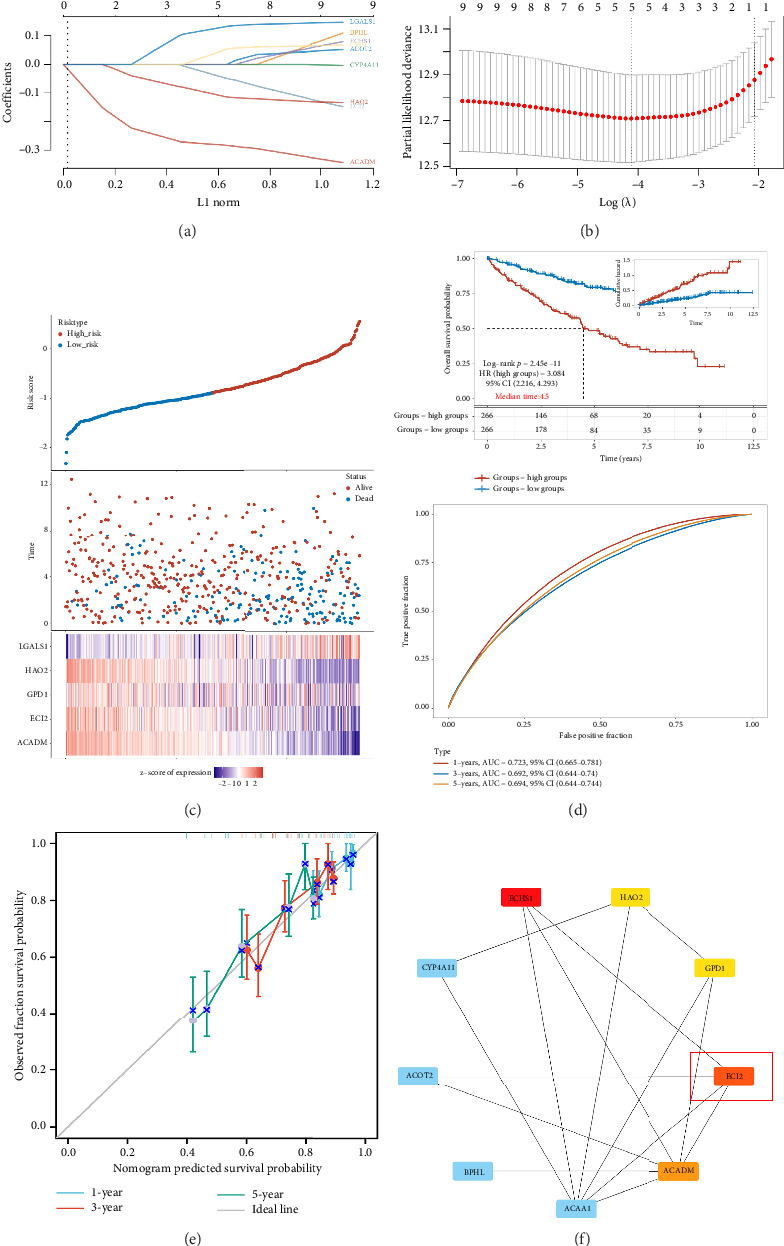
Prognostic model related to fatty acid metabolism. (a) Cross-validation plot for the penalty term. (b) Plots for LASSO expression coefficients of the FA-DEGs. (c) Risk score, survival time, and survival status of ccRCC patients in TCGA. (d) The KM survival curve distribution of this risk model in ccRCC patients, where log rank is used to test between different groups. (e) Calibration curve of fatty acid metabolism–related risk model. (f) Screening key FA-DEGs using Cytoscape.

We employed the “survival” package to conduct a proportional hazards hypothesis test on FA-DEGs and performed a Cox regression analysis. The univariate analysis revealed that nine genes possess prognostic significance, while the multivariate analysis indicated that groups with high *HAO2* and *ACADM* expression exhibit a favorable prognosis (Table 1). Next, we established a prognostic model related to fatty acid metabolism using 10 FA-DEGs. Using the LASSO algorithm for screening, a total of six core FADEGs were identified, including *ECI2, GPD1, HAO2, LGALS1*, and *ACADM*, for constructing a risk scoring prognostic model (Figures 2(a) and 2(b)). According to the LASSO risk score, patients were divided into high-risk and low-risk groups for survival prognosis analysis. The results showed that the high-risk group had a worse prognosis than the low-risk group (Figures 2(c) and 2(d)). The risk factor map also shows that the higher the LASSO risk score, the worse the prognosis. Calibration analysis shows that the actual probability fits well with the predicted probability of the model, indicating that the model has good prognostic performance (Figure 2(e)). Next, we used Cytoscape software to calculate the core differentially expressed genes in FA-DEGS, and ultimately identified six core DIRGs including *ECI2, HAO2, ACAA1, ECHS1*, and *ACADM* (Figure 2(f)). Given that other genes have been confirmed to be associated with ccRCC in previous studies [23–26], we have chosen *ECI2* as the subsequent target gene.

### 3.3. Analysis of ECI2 Expression Levels and Its Correlation With Clinical Characteristics and Prognosis

**Figure 3:**
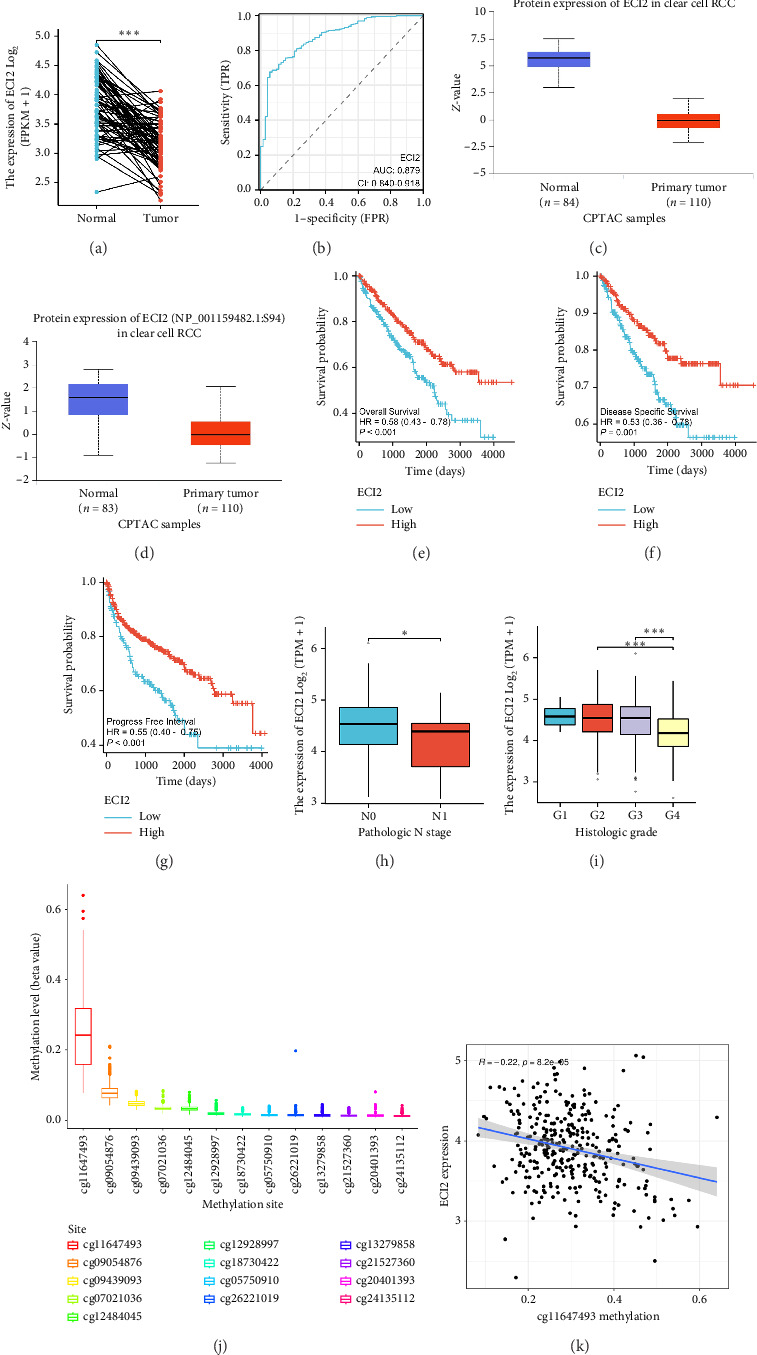
Analysis of ECI2 expression levels and its correlation with clinical characteristics and prognosis. (a) ECI2 expression in paired samples in ccRCC cohort of TCGA. (b) ROC analysis of ECI2 in ccRCC cohort diagnosis. (c) Differences protein expression levels of ECI2 in CPTAC. (d) Differences phosphorylated protein expression levels of ECI2 in CPTAC. (e) Analysis of the correlation between ECI2 expression and ccRCC patients' overall survival. (f) Analysis of the correlation between ECI2 expression and ccRCC patients' disease-specific survival. (g) Analysis of the correlation between ECI2 expression and ccRCC patients' progression-free survival. (h) Correlation analysis between the expression level of ECI2 and the pathological N stage of ccRCC patients. (i) Correlation analysis between the expression level of ECI2 histologic stage of ccRCC patients. (j) Analysis of methylation levels of ECI2 at various points. (k) Correlation analysis between methylation level and mRNA expression level of ECI2 at cg11647493 site. Data were shown as mean ± SD. ^∗^*p* < 0.05, ^∗∗^*p* < 0.01, ^∗∗∗^*p* < 0.001, ^∗∗∗∗^*p* < 0.0001.

Analysis of TCGA transcriptome data revealed that *ECI2* was significantly downregulated in various tumor tissues, including ccRCC (Figure 3(a)). The AUC curve indicates that *ECI2* provides effective performance in diagnosing ccRCC (Figure 3(b)). Subsequently, we examined the protein expression levels of *ECI2* using CPTAC data, revealing a decrease in both total and phosphorylated protein levels of *ECI2* within tumor tissues (Figures 3(c) and 3(d)). Prognostic analysis demonstrated that patients with high *ECI2* expression exhibited improved survival rates (Figures 3(e), 3(f), and 3(g)), including overall survival (OS), progression-free survival (PFS), and disease-free survival (DFS). Clinical characteristic analysis indicated that *ECI2* expression negatively correlates with pathologic N stage and histologic grade (Figures 3(h) and 3(i)). Methylation level analysis revealed that *ECI2* exhibits a high methylation level at the cg11647493 site, and the expression of *ECI2* is inversely correlated with the methylation level at this site (Figures 3(j) and 3(k)).

### 3.4. Gene Function Enrichment Analysis of *ECI2*

**Figure 4:**
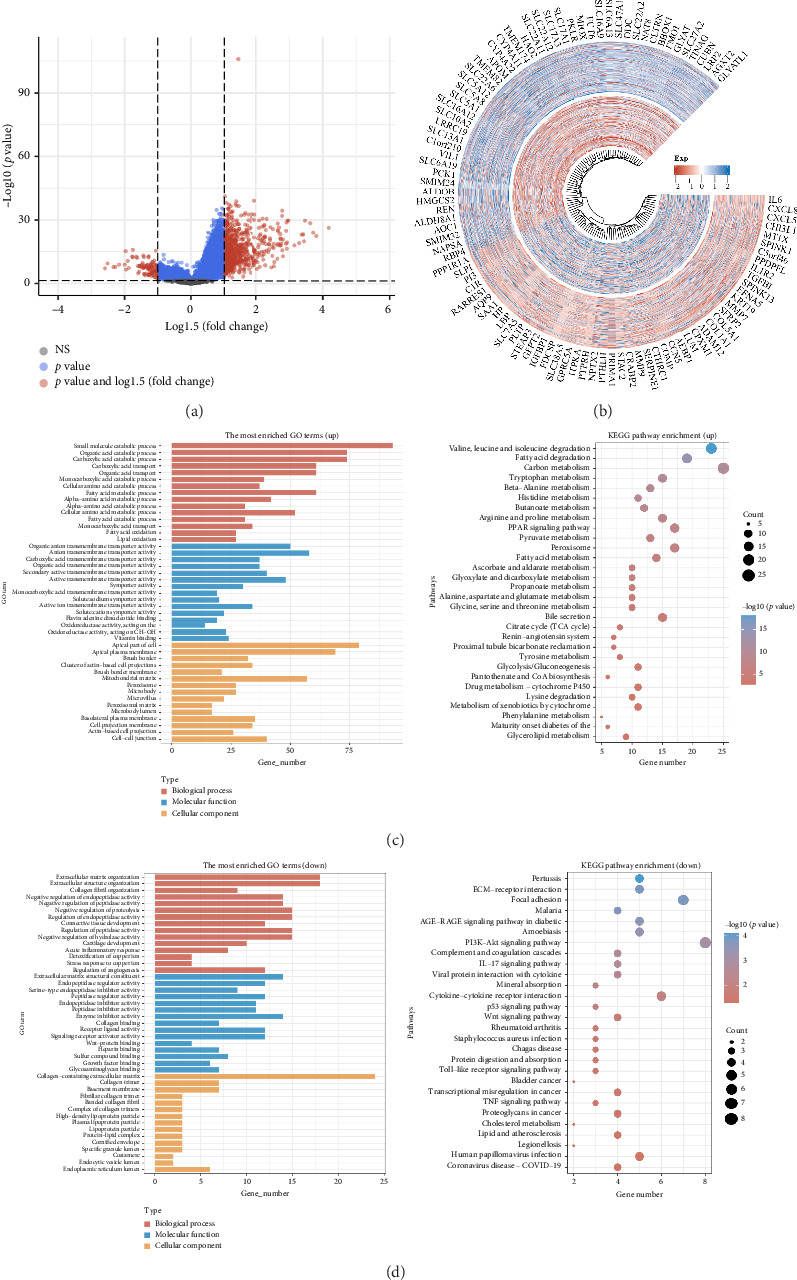
Gene function enrichment analysis of ECI2. (a) Volcano map of differentially expressed genes between high- and low-expression groups of ECI2. (b) Differential gene expression heatmap of top 100 upregulated and downregulated genes. (c) KEGG pathway and GO term enrichment results of differentially upregulated genes. (d) KEGG pathway and GO term enrichment results of differentially downregulated genes.

To further explore the gene function of *ECI2*, we identified 700 upregulated and 100 downregulated genes (Figures 4(a) and 4(b)). GO and KEGG enrichment analysis indicated that *ECI2* is positively associated with fatty acid degradation and PARP signaling pathway (Figure 4(c)) and negatively associated with the PI3K/AKT, Wnt, and TNF signaling pathway (Figure 4(d)).

### 3.5. Analysis of ECI2 and Immune Infiltration

**Figure 5:**
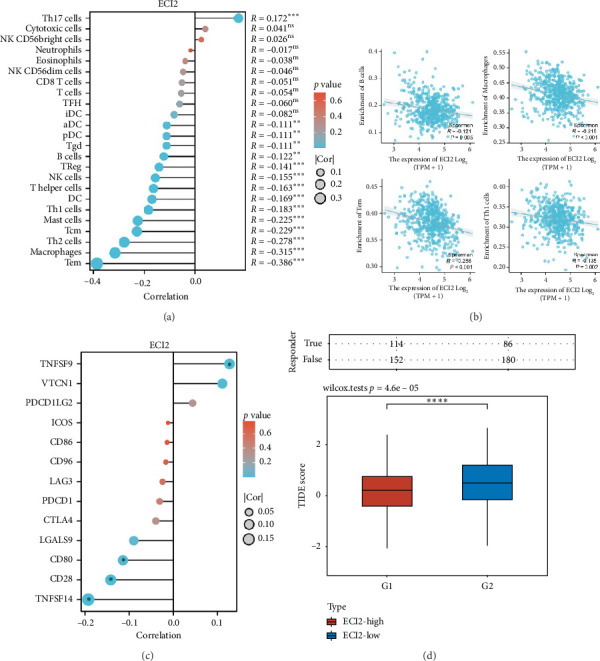
Analysis of ECI2 and immune infiltration. (a) Analysis of the correlation between ECI2 and 24 types of immune cell infiltration. (b) Analysis of the correlation between ECI2 and macrophages, B cells, Th1 cells, and TEM cells. (c) Analysis of the correlation between ECI2 and 13 immune checkpoints. (d) Distribution of TIDE scores in ECI2 high-expression group and low-expression group. Data were shown as mean ± SD. ^∗^*p* < 0.05, ^∗∗^*p* < 0.01, ^∗∗∗^*p* < 0.001, ^∗∗∗∗^*p* < 0.0001.

Using the ssGSEA algorithm from the GSVA package, we assessed the correlation between *ECI2* expression levels and immune cells. The findings revealed that *ECI2* expression negatively correlates with various immune cells in ccRCC (Figure 5(a)), including macrophages, B cells, Th1 cells, and Tem cells (Figure 5(b)). Spearman correlation analysis showed that *ECI2* was significantly negatively correlated with multiple immune checkpoints, including CD80, CD28, and TNFSF14 (Figure 5(c)). The TIDE score indicates that the group with high *ECI2* expression demonstrates enhanced responsiveness to ICB therapy and a more favorable prognosis (Figure 5(d)).

### 3.6. Expression Validation of ECI2 in Clinical Samples and Cell Lines

**Figure 6:**
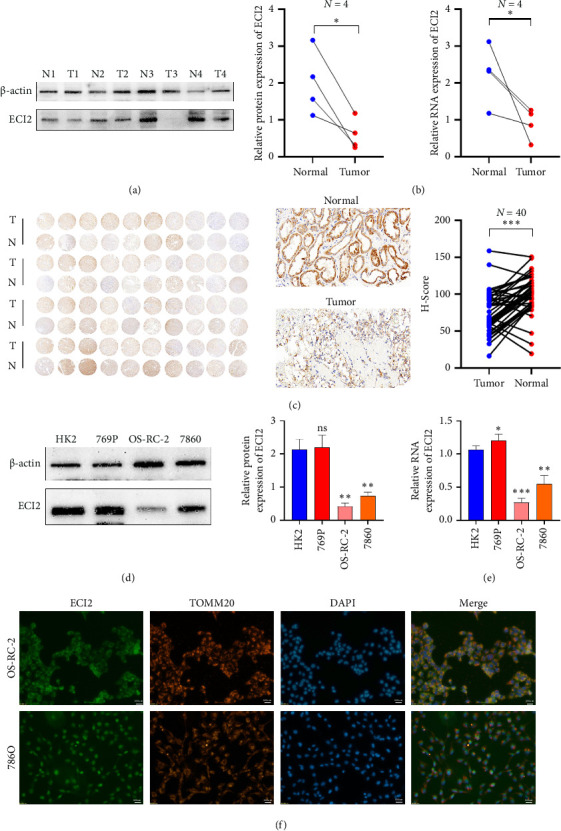
Expression validation of ECI2 in clinical samples and cell lines. (a) Differences in protein expression levels of ECI2 in ccRCC tumor tissue and adjacent tissues (*n* = 8). (b) Differences in mRNA expression levels of ECI2 in ccRCC tumor tissue and adjacent tissues (*n* = 8). (c) Immunohistochemical staining of ccRCC tumor tissue and adjacent tissues (*n* = 40). Scale bars: 200 μm. (d) Differences in protein expression levels of ECI2 in ccRCC cell lines. (e) Differences in mRNA expression levels of ECI2 in ccRCC cell lines. (f) Immunofluorescence staining confirms the subcellular localization of ECI2 (using FITC to label ECI2 and CY3 to label mitochondrial protein TOM20). Data were shown as mean ± SD. ^∗^*p* < 0.05, ^∗∗^*p* < 0.01, ^∗∗∗^*p* < 0.001, ^∗∗∗∗^*p* < 0.0001.

We collected eight pairs of ccRCC tumor samples and adjacent nontumor samples. Western blot and RT-PCR experiments demonstrated that both protein and mRNA expression levels of *ECI2* were significantly reduced in tumor tissues (Figures 6(a) and 6(b)). Immunohistochemical staining indicated that *ECI2* had lower scores in around 80% of tumor samples (Figure 6(c)). Subsequently, we assessed the differential expression of *ECI2* across ccRCC tumor cell lines, revealing significant reductions in the 786O and OS-RC-2 cell lines (Figures 6(d) and 6(e)). Using TOM20 to label mitochondria, immunofluorescence staining suggested that *ECI2* may reside within the mitochondria (Figure 6(f)).

### 3.7. Overexpression of *ECI2* Inhibits the Proliferation and Migration of ccRCC Cells

**Figure 7:**
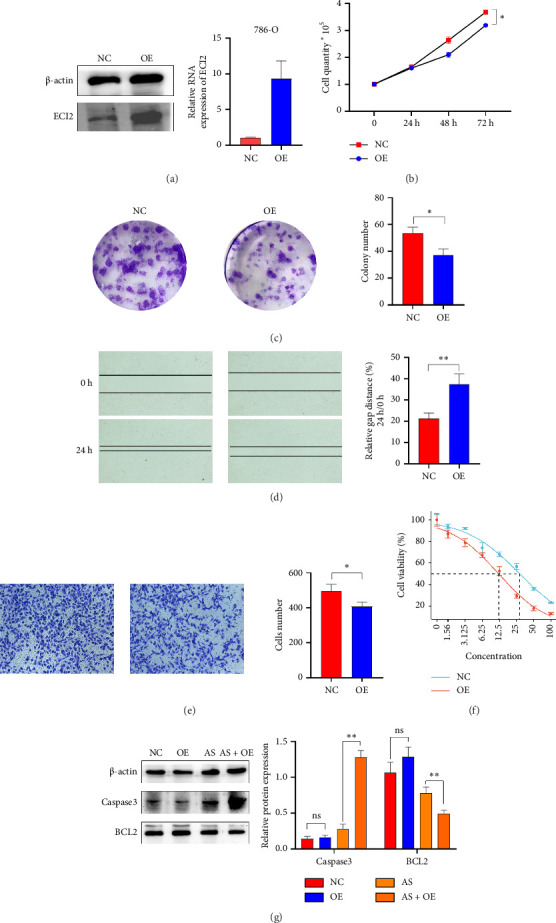
Overexpression of ECI2 inhibits the proliferation and migration of ccRCC cells. (a) Differences in protein and mRNA expression levels of TBRG4 after lentiviral transfection into 786O. (b) The proliferation of 786O was examined by cell counts. (c) Colony formation assay was employed to evaluate the proliferation of 786O. (d) Wound healing assay measured the motor ability of 786O. (e) The transwell assay detected the migration of 786O. (f) Calculation of IC_50_ differences after 48 h of treatment with different concentrations of oxaliplatin. (g) Western blot detection of protein expression differences between BCL2 and Caspase3 after 48 h of IC_50_ oxaliplatin treatment. Data were shown as mean ± SD. ^∗^*p* < 0.05, ^∗∗^*p* < 0.01, ^∗∗∗^*p* < 0.001, ^∗∗∗∗^*p* < 0.0001.

To investigate the impact of *ECI2* on the function of ccRCC cells, we transfected 786O cells with a lentivirus expressing an *ECI2* overexpression plasmid. Western blot and RT-PCR confirmed the overexpression of *ECI2* (Figure 7(a)). Growth curve and colony formation assays indicated that overexpression of *ECI2* suppressed the proliferation of 786O cells (Figures 7(b) and 7(c)). Scratch and Transwell experiments demonstrated that *ECI2* inhibits the migration of 786O cells (Figures 7(d) and 7(e)). Additionally, after treating 786O cells with various concentrations of oxaliplatin, we observed that overexpression of *ECI2* reduced the IC_50_, thereby enhancing the drug sensitivity of 786O cells (Figure 7(f)). Subsequently, we treated the cells with oxaliplatin at the IC50 concentration for 48 h and then collected them. Western blot analysis indicated that overexpression of *ECI2* significantly elevated the expression level of Caspase3 induced by oxaliplatin treatment and concurrently reduced the expression level of BCL2 (Figure 7(g)).

## 4. Discussion

As the most prevalent subtype of RCC, ccRCC is marked by metabolic reprogramming, with abnormal alterations in fatty acid metabolism significantly influencing tumor progression. Fatty acid metabolism encompasses fatty acid synthesis, uptake, and FAO, pivotal roles in lipid storage and metabolism [27]. Fatty acids within lipid droplets act as substrates for lipid synthesis, curbing the release of lipid-derived reactive oxygen species and thereby mitigating lip toxicity. Additionally, they undergo FAO in mitochondria to produce energy [28]. Targeting key genes in fatty acid metabolism demonstrates substantial antitumor effects, with certain potential therapeutic targets already advancing to clinical trials [29, 30]. Our study reveals a general downward trend in the expression of the fatty acid metabolism gene set within ccRCC tumor tissues, suggesting that certain key genes may decrease lipid droplet accumulation in ccRCC cells via the regulation of fatty acid metabolism.

By employing proteomics, TCGA transcriptomics, and single-cell differential gene analysis, we pinpointed 10 key genes with differential expression, nine of which were downregulated and one upregulated. To explore the association between these genes and the initiation and progression of ccRCC, we developed a prognostic risk prediction model utilizing five genes through LASSO and multivariate Cox regression analysis. Furthermore, a calibration curve was constructed to evaluate the concordance between the predicted risk from the model and the actual risk. The findings suggest that the model demonstrates robust predictive abilities for the prognosis of ccRCC patients.

Five key genes were identified using Cytoscape software. Given the scarcity of research on ECI2's role in ccRCC, we selected *ECI2* as our subsequent research focus. *ECI2* is pivotal in fatty acid metabolism, balancing lipid metabolism both inside and outside cells, and ensuring normal cellular energy metabolism. Studies indicate that *ECI2* serves as a reliable prognostic biomarker in both ovarian and colon cancers [31, 32] and promotes tumor cell proliferation in prostate cancer [33, 34]. Our study reveals that *ECI2* levels are markedly reduced in ccRCC, correlating with a favorable prognosis for patients. Additionally, a significant increase in *ECI2* methylation at cg11647493 may contribute to the reduced levels of *ECI2* during the progression of ccRCC. Functional enrichment analysis indicates that *ECI2* is inversely associated with several tumor proliferation signaling pathways, including PI3K/AKT, Wnt, and TNF signaling pathways. To explore the impact of *ECI2* on ccRCC tumor cells, we developed a cell line overexpressing *ECI2*. The findings indicate that overexpression of *ECI2* suppresses tumor cell growth and migration and enhances ccRCC cells' sensitivity to oxaliplatin.

Because kidney cancer typically does not respond well to chemotherapy, it is not a standard treatment option. While certain chemotherapy agents such as cisplatin, 5-fluorouracil (5-FU), and gemcitabine have shown efficacy in a subset of kidney cancer patients [35], the majority of cases are primarily treated with surgery. Drug toxicity tests have demonstrated that overexpressing *ECI2* enhances the sensitivity of kidney cancer cells to oxaliplatin and elevates the expression of Caspase3 induced by oxaliplatin. *ECI2* could serve as a drug target to inform clinical medication decisions.

In conclusion, our study assessed the expression levels of the fatty acid metabolism gene set in ccRCC and developed a prognostic model for fatty acid metabolism that demonstrates strong predictive capabilities. Additionally, we conducted an in-depth exploration of *ECI2*'s differential expression and functional analysis and verified the impact of *ECI2* overexpression on the functionality of ccRCC tumor cells. However, the precise mechanisms require further investigation.

## 5. Limitation

Our study's reliance on retrospective data and in vitro findings may limit the generalizability and clinical applicability of the results, underscoring the need for validation in larger cohorts and in vivo models. Further investigation into ECI2's regulatory mechanisms and involvement in broader metabolic pathways is essential to confirm its therapeutic potential in ccRCC.

## Figures and Tables

**Table 1:** Univariate and multivariate prognostic analyses of FA-DEGs.

Characteristics	Total (*N*)	HR (95% CI) univariate analysis	*p* value univariate analysis	HR (95% CI) multivariate analysis	*p* value multivariate analysis
ECI2	541				
Low	270	Reference		Reference	
High	271	0.517 (0.381–0.703)	< 0.001	0.812 (0.562–1.174)	0.268
ACAA1	541				
Low	270	Reference		Reference	
High	271	0.524 (0.385–0.715)	< 0.001	0.943 (0.646–1.376)	0.760
GPD1	541				
Low	270	Reference			
High	271	0.851 (0.632–1.145)	0.285		
HAO2	541				
Low	270	Reference		Reference	
High	271	0.438 (0.321–0.599)	< 0.001	0.646 (0.430–0.970)	0.035
CYP4A11	541				
Low	270	Reference		Reference	
High	271	0.584 (0.432–0.790)	< 0.001	1.125 (0.755–1.677)	0.562
LGALS1	541				
Low	270	Reference		Reference	
High	271	1.357 (1.006–1.829)	0.045	0.990 (0.718–1.365)	0.949
ACOT2	541				
Low	270	Reference		Reference	
High	271	0.709 (0.525–0.956)	0.024	1.093 (0.782–1.528)	0.603
ECHS1	541				
Low	270	Reference		Reference	
High	271	0.611 (0.452–0.826)	0.001	1.110 (0.772–1.597)	0.572
ACADM	541				
Low	270	Reference		Reference	
High	271	0.319 (0.229–0.445)	< 0.001	0.399 (0.259–0.613)	< 0.001
BPHL	541				
Low	270	Reference		Reference	
High	271	0.463 (0.338–0.635)	< 0.001	0.919 (0.607–1.392)	0.691

*Note:* Data were shown as mean ± SD.

^∗^ *p* < 0.05.

^∗∗^ *p* < 0.01.

^∗∗∗^ *p* < 0.001.

^∗∗∗∗^ *p* < 0.0001.

## Data Availability

All data included in this study are available upon request by contact with the corresponding author.

## Supporting Information

Western blot original imprint image.

## References

[1] Ljungberg B., Albiges L., Abu-Ghanem Y. (2022). European Association of Urology Guidelines on Renal Cell Carcinoma: The 2022 Update. *European Urology*.

[2] Patard J. J., Leray E., Rioux-Leclercq N. (2005). Prognostic Value of Histologic Subtypes in Renal Cell Carcinoma: A Multicenter Experience. *Journal of Clinical Oncology*.

[3] Lee W. K., Hong S. K., Lee S. (2015). Prognostic Value of Body Mass Index According to Histologic Subtype in Nonmetastatic Renal Cell Carcinoma: A Large Cohort Analysis. *Clinical Genitourinary Cancer*.

[4] Siegel R. L., Miller K. D., Wagle N. S., Jemal A. (2023). Cancer Statistics, 2023. *CA: A Cancer Journal for Clinicians*.

[5] Hsieh J. J., Purdue M. P., Signoretti S. (2017). Renal Cell Carcinoma. *Nature Reviews Disease Primers*.

[6] Leibovich B. C., Lohse C. M., Cheville J. C. (2018). Predicting Oncologic Outcomes in Renal Cell Carcinoma After Surgery. *European Urology*.

[7] Bian X., Liu R., Meng Y., Xing D., Xu D., Lu Z. (2021). Lipid Metabolism and Cancer. *The Journal of experimental medicine*.

[8] Cheng C., Geng F., Cheng X., Guo D. (2018). Lipid Metabolism Reprogramming and Its Potential Targets in Cancer. *Cancer Communications*.

[9] Heravi G., Yazdanpanah O., Podgorski I., Matherly L. H., Liu W. (2022). Lipid Metabolism Reprogramming in Renal Cell Carcinoma. *Cancer and Metastasis Reviews*.

[10] Du W., Zhang L., Brett-Morris A. (2017). HIF Drives Lipid Deposition and Cancer in ccRCC via Repression of Fatty Acid Metabolism. *Nature Communications*.

[11] Drabkin H. A., Gemmill R. M. (2012). Cholesterol and the Development of Clear-Cell Renal Carcinoma. *Current Opinion in Pharmacology*.

[12] Fan J., Li X., Issop L., Culty M., Papadopoulos V. (2016). ACBD2/ECI2-Mediated Peroxisome-Mitochondria Interactions in Leydig Cell Steroid Biosynthesis. *Molecular Endocrinology*.

[13] Geisbrecht B. V., Zhang D., Schulz H., Gould S. J. (1999). Characterization of PECI, a Novel Monofunctional Δ3,Δ2-Enoyl-CoA Isomerase of Mammalian Peroxisomes. *Journal of Biological Chemistry*.

[14] Su C., Lv Y., Lu W. (2021). Single-Cell RNA Sequencing in Multiple Pathologic Types of Renal Cell Carcinoma Revealed Novel Potential Tumor-Specific Markers. *Frontiers in Oncology*.

[15] Satija R., Farrell J. A., Gennert D., Schier A. F., Regev A. (2015). Spatial Reconstruction of Single-Cell Gene Expression Data. *Nature Biotechnology*.

[16] Butler A., Hoffman P., Smibert P., Papalexi E., Satija R. (2018). Integrating Single-Cell Transcriptomic Data across Different Conditions, Technologies, and Species. *Nature Biotechnology*.

[17] Friedman J., Hastie T., Tibshirani R. (2010). Regularization Paths for Generalized Linear Models via Coordinate Descent. *Journal of Statistical Software*.

[18] Diboun I., Wernisch L., Orengo C. A., Koltzenburg M. (2006). Microarray Analysis after RNA Amplification Can Detect Pronounced Differences in Gene Expression Using Limma. *BMC Genomics*.

[19] The Gene Ontology Consortium (2015). Gene Ontology Consortium: Going Forward. *Nucleic Acids Research*.

[20] Yu G., Wang L. G., Han Y., He Q. Y. (2012). clusterProfiler: An R Package for Comparing Biological Themes Among Gene Clusters. *OMICS: A Journal of Integrative Biology*.

[21] Jiang P., Gu S., Pan D. (2018). Signatures of T Cell Dysfunction and Exclusion Predict Cancer Immunotherapy Response. *Nature Medicine*.

[22] Cui D., Cui X., Xu X. (2022). Identification of TLN1 as a Prognostic Biomarker to Effect Cell Proliferation and Differentiation in Acute Myeloid Leukemia. *BMC Cancer*.

[23] Zhou L., Yin M., Guo F., Yu Z., Weng G., Long H. (2024). Low ACADM Expression Predicts Poor Prognosis and Suppressive Tumor Microenvironment in Clear Cell Renal Cell Carcinoma. *Scientific Reports*.

[24] Qu Y. Y., Zhao R., Zhang H. L. (2020). Inactivation of the AMPK-GATA3-ECHS1 Pathway Induces Fatty Acid Synthesis that Promotes Clear Cell Renal Cell Carcinoma Growth. *Cancer Research*.

[25] Xiao W., Wang X., Wang T., Chen B., Xing J. (2019). HAO2 Inhibits Malignancy of Clear Cell Renal Cell Carcinoma by Promoting Lipid Catabolic Process. *Journal of Cellular Physiology*.

[26] Liu R., Feng Y., Deng Y. (2021). A HIF1α-GPD1 Feedforward Loop Inhibits the Progression of Renal Clear Cell Carcinoma via Mitochondrial Function and Lipid Metabolism. *Journal of Experimental & Clinical Cancer Research*.

[27] Tan S. K., Mahmud I., Fontanesi F. (2021). Obesity-Dependent Adipokine Chemerin Suppresses Fatty Acid Oxidation to Confer Ferroptosis Resistance. *Cancer Discovery*.

[28] Fucho R., Casals N., Serra D., Herrero L. (2017). Ceramides and Mitochondrial Fatty Acid Oxidation in Obesity. *The FASEB Journal*.

[29] Manson J. E., Cook N. R., Lee I. M. (2019). Marine N-3 Fatty Acids and Prevention of Cardiovascular Disease and Cancer. *New England Journal of Medicine*.

[30] Sun G., Davies J. R., Mell T. (2024). APOE Genotype, Eicosapentaenoic Acid (EPA) Supplementation and N-3 Highly Unsaturated Fatty Acid (HUFA) Levels in Patients With Multiple Colorectal Polyps: A Secondary Analysis of the seAFOod Polyp Prevention Trial. *Prostaglandins, Leukotrienes and Essential Fatty Acids*.

[31] Meng C., Sun Y., Liu G. (2023). Establishment of a Prognostic Model for Ovarian Cancer Based on Mitochondrial Metabolism-Related Genes. *Frontiers in Oncology*.

[32] Cheraghi-Shavi T., Jalal R., Minuchehr Z. (2023). TGM2, HMGA2, FXYD3, and LGALS4 Genes as Biomarkers in Acquired Oxaliplatin Resistance of Human Colorectal Cancer: A Systems Biology Approach. *PLoS One*.

[33] Dan C., Zhang H., Zeng W. (2019). HNF1B Expression Regulates ECI2 Gene Expression, Potentially Serving a Role in Prostate Cancer Progression. *Oncology Letters*.

[34] Itkonen H. M., Brown M., Urbanucci A. (2017). Lipid Degradation Promotes Prostate Cancer Cell Survival. *Oncotarget*.

[35] Makhov P., Joshi S., Ghatalia P., Kutikov A., Uzzo R. G., Kolenko V. M. (2018). Resistance to Systemic Therapies in Clear Cell Renal Cell Carcinoma: Mechanisms and Management Strategies. *Molecular Cancer Therapeutics*.

