# Supplementary figures and images for: Identification of ECI2 as Potential Prognostic Biomarkers Based on a Fatty Acid Metabolism-Related Gene Model in Clear Cell Renal Cell Carcinoma
Source: Genet Res (Camb). 2025 May 19;2025:2237539. doi: 10.1155/genr/2237539 (PMC12105886; doi:10.1155/genr/2237539)

Figure6A

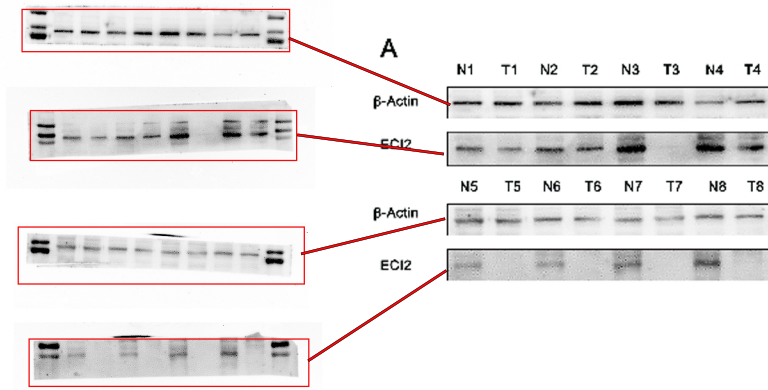

Figure7A

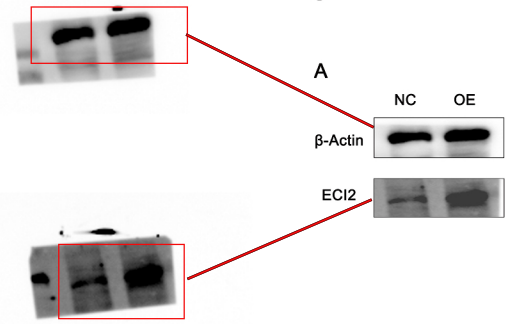

Figure6D

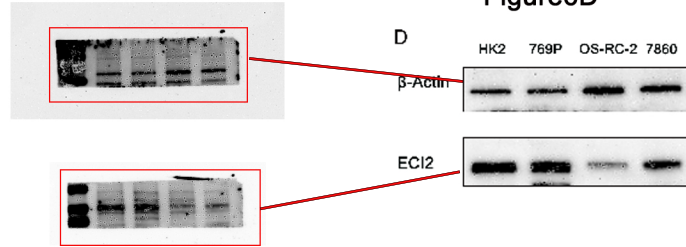

Figure7G

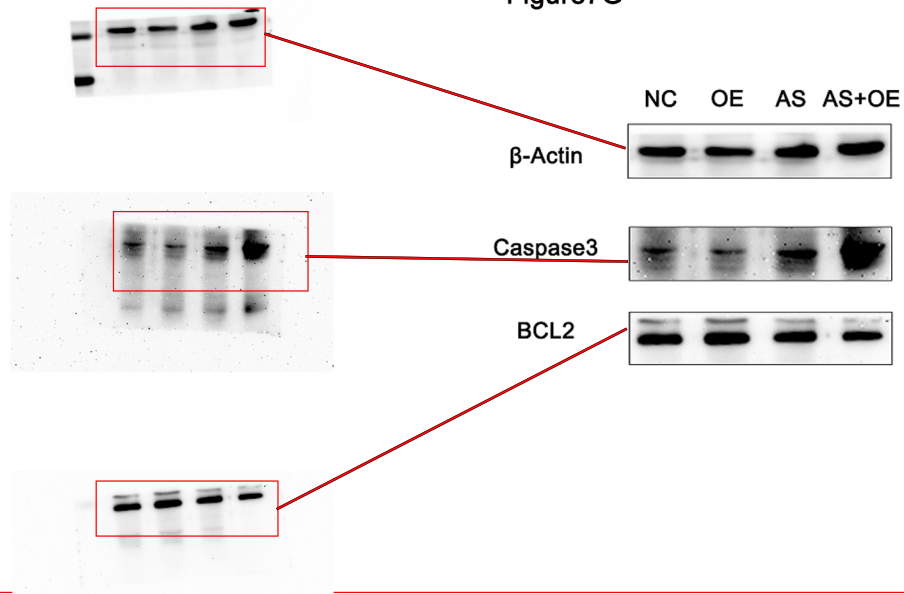

Supplement: Supporting Information — Additional supporting information can be found online in the Supporting Information section. [file 2237539.f1.pdf]
